# Can air pollution negate the health benefits of cycling and walking?

**DOI:** 10.1016/j.ypmed.2016.02.002

**Published:** 2016-06

**Authors:** Marko Tainio, Audrey J. de Nazelle, Thomas Götschi, Sonja Kahlmeier, David Rojas-Rueda, Mark J. Nieuwenhuijsen, Thiago Hérick de Sá, Paul Kelly, James Woodcock

**Affiliations:** a UKCRC Centre for Diet and Activity Research, MRC Epidemiology Unit, University of Cambridge School of Clinical Medicine, Institute of Metabolic Science, Cambridge, UK; b Centre for Environmental Policy, Imperial College London, London, UK; c Physical Activity and Health Unit, Epidemiology, Biostatistics and Prevention Institute, University of Zurich, Zurich, Switzerland; d Center for Research in Environmental Epidemiology (CREAL), Barcelona, Spain; e Universitat Pompeu Fabra (UPF), Barcelona, Spain; f Centro de Investigación Biomédica en Red de Epidemiología y Salud Pública (CIBERESP), Madrid, Spain; g Centre for Epidemiological Research in Nutrition and Health, School of Public Health, University of São Paulo, São Paulo, Brazil; h Physical Activity for Health Research Centre (PAHRC), University of Edinburgh, UK

**Keywords:** Physical activity, Air pollution, Bicycling, Walking, Mortality, Health Impact Assessment, Risk–Benefit Assessment

## Abstract

Active travel (cycling, walking) is beneficial for the health due to increased physical activity (PA). However, active travel may increase the intake of air pollution, leading to negative health consequences. We examined the risk–benefit balance between active travel related PA and exposure to air pollution across a range of air pollution and PA scenarios.

The health effects of active travel and air pollution were estimated through changes in all-cause mortality for different levels of active travel and air pollution. Air pollution exposure was estimated through changes in background concentrations of fine particulate matter (PM_2.5_), ranging from 5 to 200 μg/m3. For active travel exposure, we estimated cycling and walking from 0 up to 16 h per day, respectively. These refer to long-term average levels of active travel and PM_2.5_ exposure.

For the global average urban background PM_2.5_ concentration (22 μg/m3) benefits of PA by far outweigh risks from air pollution even under the most extreme levels of active travel. In areas with PM_2.5_ concentrations of 100 μg/m3, harms would exceed benefits after 1 h 30 min of cycling per day or more than 10 h of walking per day. If the counterfactual was driving, rather than staying at home, the benefits of PA would exceed harms from air pollution up to 3 h 30 min of cycling per day. The results were sensitive to dose–response function (DRF) assumptions for PM_2.5_ and PA.

PA benefits of active travel outweighed the harm caused by air pollution in all but the most extreme air pollution concentrations.

## Highlights

• Air pollution (AP) may reduce the health benefits of active travel (AT).
• We compared risk–benefit trade-off of AP and physical activity (PA) due to AT.
• In most urban environments benefits of PA outweighed risks of AP.
• If cycling replaces driving, the trade-off would be even more beneficial.

## Introduction

Several health impact modelling (HIM) studies have estimated the health benefits and risks of active travel (cycling, walking) in different geographical areas (Mueller et al., 2015, Doorley et al., 2015). In most of these studies, the health benefits due to physical activity (PA) from increased active travel are significantly larger than the health risks caused by increases in exposure to air pollution.

Most of the existing active travel HIM studies have been carried out in cities in high income countries with relatively low air pollution levels (Mueller et al., 2015, Doorley et al., 2015). This raises the question on the risk–benefit balance in highly polluted environments. Health risks of air pollution are usually thought to increase linearly with increased exposure for low to moderate levels of air pollution, whereas the benefits of PA increase curvy-linearly with increasing dose (Kelly et al., 2014, World Health Organization, 2014). Thus, at a certain level of background air pollution and of active travel, risks could outweigh benefits, which would directly imply that, from a public health perspective, active travel could not be always recommended.

**Fig. 1:**
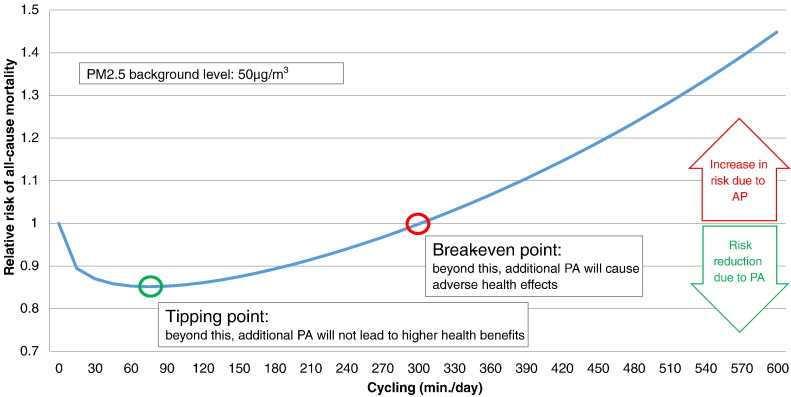
Illustration of tipping point and break-even point as measured by the relative risk (RR) for all-cause mortality (ACM) combining the effects of air pollution (at 50 μg/m^3^ PM_2.5_) and physical activity (cycling).

In this study we compare the health risks of air pollution with the PA-related health benefits from active travel across a wide range of possible air pollution concentrations and active travel levels. We use two thresholds to compare PA benefits and air pollution risks (Fig. 1): At the “*tipping point*” an incremental increase in active travel will no longer lead to an increase in health benefits (i.e. max. benefits have been reached). Increasing active travel even more could lead to the “*break-even point*”, where risk from air pollution starts outweighing the benefits of PA (i.e. there are no longer net benefits, compared to not engaging in active travel).

## Methods

Our approach followed a general active travel HIM method (Mueller et al., 2015, Doorley et al., 2015). Air pollution exposures due to active travel were quantified by estimating the differences in the inhaled dose of fine particulate matter (PM_2.5_) air pollution. We selected PM_2.5_ because it is a commonly used indicator of air pollution in active travel HIM studies (Mueller et al., 2015, Doorley et al., 2015), and because of the large health burden caused by PM_2.5_ (GBD 2013 Risk Factors Collaborators et al., 2015). For both air pollution and PA we used all-cause mortality as the health outcome because there is strong evidence for its association with both long-term exposure to PM_2.5_ (Héroux et al., 2015) and long-term PA behaviour (Kelly et al., 2014).

The reduction in all-cause mortality from active travel was estimated by converting the time spent cycling or walking to metabolically equivalent of task (MET) and calculating the risk reduction using dose–response functions (DRFs) adapted from Kelly et al.'s^3^ meta-analysis. From the different DRFs reported in Kelly et al. (2014) we chose the one with the “0.50 power transformation” as a compromise between linear and extremely non-linear DRFs. Non-linearity in a DRF means that the health benefits of increased active travel would level out sooner and a tipping point would be reached earlier than with more linear DRFs. See supplementary material for the sensitivity analysis with different DRFs. To convert cycling and walking time to PA we used the values of 4.0 METs for walking and 6.8 METs for cycling, based on the Compendium of Physical Activities (Ainsworth et al., 2011). The walking and cycling levels used in this study are assumed to reflect long-term average behaviour.

The health risks of PM_2.5_ were estimated by converting background PM_2.5_ concentrations to travel mode specific exposure concentrations, and by taking into account ventilation rate whilst being active. For background PM_2.5_ we used values between 5 and 200 μg/m3 with 5 μg/m3 intervals. We also estimated tipping points and break-even points for the average and most polluted cities in each region included in the World Health Organization (WHO) Ambient Air Pollution Database (World Health Organization (WHO), 2014), which contains measured and estimated background PM_2.5_ concentrations for 1622 cities around the world.

The mode specific exposure concentrations were estimated by multiplying background PM_2.5_ concentration by 2.0 for cycling or 1.1 for walking, based on a review of studies (Kahlmeier et al., 2014). The counterfactual scenario for the time spent cycling or walking was assumed to be staying at home (i.e. in background concentration of PM_2.5_). See supplementary file for the sensitivity analysis with counterfactual scenarios where cycling time would replace motorised transport time. The ventilation rates differences whilst at sleep, rest, cycling and walking were taken into account when converting exposure to inhaled dose. For sleep, rest, walking and cycling we used ventilation rates of 0.27, 0.61, 1.37 and 2.55, respectively (de Nazelle et al., 2009, Johnson, 2002). The sleep time was assumed to be 8 h in all scenarios and the resting time was 16 h minus the time for active travel.

For the PM_2.5_ DRF we used a relative risk (RR) value of 1.07 per 10 μg/m3 change in exposure (World Health Organization, 2014). We assumed that DRF is linear from zero to maximum inhaled dose. As a sensitivity analysis we used non-linear integrated risk function from Burnett et al. (2014) (see supplementary material for details).

The model used for all calculations is provided in Lumina Decision Systems Analytica format in supplementary file 2 (readable with Analytica Free 101, http://www.lumina.com/products/free101/), and a simplified model containing the main results is provided in Microsoft Excel format in supplementary file 3.

## Results

**Fig. 2:**
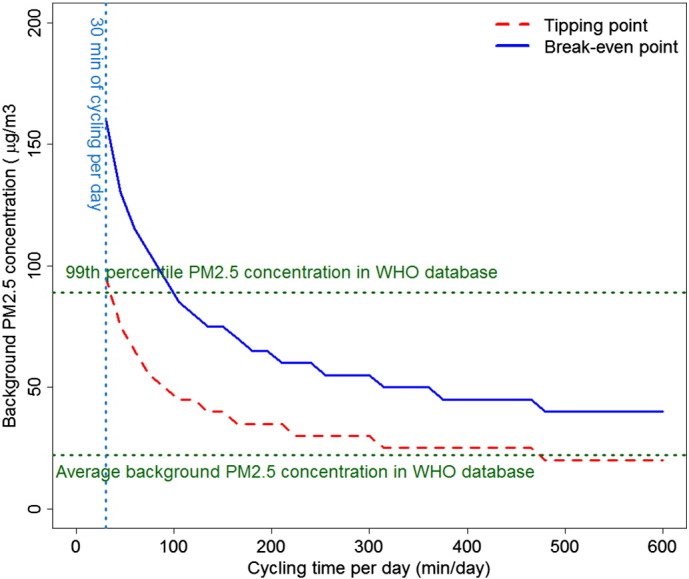
Tipping and break-even points for different levels of cycling (red dashed line and blue solid line, respectively) (minutes per day, x-axis) and for different background PM_2.5_ concentrations (y-axis). Green lines represent the average and 99th percentile background PM_2.5_ concentrations in World Health Organization (WHO) Ambient Air Pollution Database (World Health Organization (WHO), 2014).

The tipping point and break-even point for different average cycling times and background PM_2.5_ concentrations are shown in Fig. 2. For half an hour of cycling every day, the background PM_2.5_ concentration would need to be 95 μg/m3 to reach the tipping point. In the WHO Ambient Air Pollution Database less than 1% of cities have PM_2.5_ annual concentrations above that level (World Health Organization (WHO), 2014). The break-even point for half an hour of cycling every day was at 160 μg/m3 (Fig. 2). For half an hour of walking the tipping point and break-even point appear at a background concentration level above 200 μg/m3 (Fig. S3, supplementary file). For the average urban background PM_2.5_ concentration (22 μg/m3) in the WHO database, the tipping point would only be reached after 7 h of cycling and 16 h of walking per day.

Tables S2 and S3 (supplementary file) show the tipping point for cycling and walking, respectively, in different regions of the world. In the most polluted city in the database (Delhi, India, background concentration of 153 μg/m3), the tipping and break-even points were 30 and 45 min of cycling per day, respectively (Table S2, supplementary file). In most global regions the tipping points for the most polluted cities (44 μg/m3 to 153 μg/m3) varied between 30 and 120 min per day for cycling, and 90 min to 6 h 15 min per day for walking (Table S3, supplementary material).

When we assumed that time spend cycling would replace time driving a car, benefits always exceeded the risks in the background air pollution concentrations below 80 µg/m3, a concentration exceeded in only 2% of cities (World Health Organization (WHO), 2014). Other sensitivity analyses showed that the results are sensitive to the shape of the DRF functions. With the linear DRF for active travel the break-even point would be reached with background PM_2.5_ concentrations of 170 μg/m3 regardless of the active travel time (Fig. S4, supplementary material); a level not currently found in any of the cities in the WHO air pollution database (World Health Organization (WHO), 2014). With the most curved DRF (0.25 power) the PM_2.5_ concentration where harms exceed benefits for 1 h of cycling per day would drop from 150 μg/m3 to 130 μg/m3 (Fig. S4, supplementary material), a level currently found only in 9 cities (World Health Organization (WHO), 2014). With a non-linear DRF for PM_2.5_ the break-even point was not reached in any background PM_2.5_ concentration when using “power 0.50” DRF for cycling and walking. Other input value modifications had small or insignificant impact to the results.

## Discussions

This study indicates that, practically, air pollution risks will not negate the health benefits of active travel in urban areas in the vast majority of settings worldwide. Even in areas with high background PM_2.5_ concentrations, such as 100 μg/m3, up to 1 h 15 min of cycling and 10 h 30 min of walking per day will lead to net reduction in all-cause mortality (Fig. S5, supplementary material). This result is supported by epidemiological studies that have found the statistically significant protective effects of PA even in high air pollution environments (Matthews et al., 2007, Andersen et al., 2015). However, a small minority engaging in unusually high levels of active travel (i.e. bike messengers) in extremely polluted environments may be exposed to air pollution such that it negates the benefits of PA.

Some considerations of the limitations and the strengths of our study need to be applied when generalising these findings.

In this analysis we took into account only the long-term health consequences of regular PA and chronic exposure to PM_2.5_. Impacts of short-term air pollution episodes, where concentrations significantly exceed the average air pollution levels for a few days, may induce additional short term health effects. We have also only worked with all-cause mortality and have, thus, not taken into account the morbidity impact.

For the health risks of air pollution we only estimated the increased risk during cycling and walking, not the overall health risk from everyday air pollution. Air pollution causes a large burden of diseases all over the world (Burnett et al., 2014) and reducing air pollution levels would provide additional health benefits. Since transport is an important source of air pollution in urban areas, mode shifts from motorised transport to active travel would not only improve health in active travellers, but also help to reduce air pollution exposures for the whole population (Johan de Hartog et al., 2010).

The results are sensitive to assumptions of the linearity of dose–response relationships between active travel-related PA and health benefits, and between PM_2.5_ and adverse health effects. With linear DRFs for PA the benefits always exceeded the risks at all levels of PM_2.5_ concentrations. Evidence for a linear DRF for high PM_2.5_ concentrations is small and, for example, the Global Burden of Disease study applied non-linear, disease specific DRFs for PM_2.5_ (Burnett et al., 2014). If the risks of PM_2.5_ level out after PM_2.5_ concentrations over 100 μg/m3, the health benefits of PA would always exceed the risks of PM_2.5_.

It should also be taken into account that the results are based on generally representative values without detailed information on local conditions, or from the background PA and disease history of individuals. For individuals highly active in non-transport domains the benefits from active travel will be smaller, and vice versa.

## Conclusions

The benefits from active travel generally outweigh health risks from air pollution and therefore should be further encouraged. When weighing long-term health benefits from PA against possible risks from increased exposure to air pollution, our calculations show that promoting cycling and walking is justified in the vast majority of settings, and only in a small number of cities with the highest PM_2.5_ concentration in the world cycling could lead to increase in risk.

## Author contributions

MT made the calculations and drafted the first version of the manuscript. AJN, TG, MJN, SK, THS, DRR, PK and JW participated in designing the scope of the study. AJN and TG helped to clarify the message of the study. All authors contributed to the writing of this paper. All authors approved the final version to be submitted for consideration of publication.

## Conflict of interest statement

The authors declare that there are no conflicts of interests.

## Transparency document

Transparency document.

## Appendix A Supplementary data

Additional methods, tables and figures.

Analytica model that was used to calculate the results. Model can be viewed with Analytica Free 101 (http://www.lumina.com/products/free101/).

A simplified model in Microsoft Excel formal, containing the main results.
