# Supplementary material for: Can air pollution negate the health benefits of cycling and walking?
Source: Prev Med. 2016 Jun;87:233–6. doi: 10.1016/j.ypmed.2016.02.002 (PMC4893018; doi:10.1016/j.ypmed.2016.02.002)
Supplement: Supplementary file 2 — Additional methods, tables and figures. [file mmc2.docx]

**Supplementary data**

**Tainio et al. Can air pollution negate the health benefits of cycling and walking?**

**Methods - sensitivity analyses**

Shape of dose-response function (DRF) for cycling and walking

In the main analyses we assumed the “power 0.50” shape for DRFs for cycling and walking as a compromise between linear and extremely non-linear DRFs. As a sensitivity analysis we also ran calculations with “log-linear” and “0.25-power transformed” DRFs. See Figure S1 below for illustration of different DRFs for cycling, and their impact to all-cause mortality.

*Figure S1: Different transformations for dose-response function (DRF) for cycling. “Power 0.50” was the main DRF used in the analysis. DRFs are adopted from (Kelly et al. 2014).*

Air pollution adjusted DRF’s

Studies examining the health benefits of physical activity (PA) underestimate the benefits because the participants of these studies are exposed to local air pollution. Kelly et al., previously calculated pooled relative risks for walking and cycling using random-effects meta-analysis of risk estimates at 11.25 MET.hrs/week from included prospective cohort studies. Rojas Rueda (2014 – unpublished work) adjusted the risk estimates for each cohort study by estimating air pollution (PM2.5) exposure in each risk group. We re-calculated an air-pollution adjusted pooled relative risk for walking and cycling using random-effects meta-analysis of these adjusted risk estimates. See Table S1 (below) for comparison of adjusted and non-adjusted DRFs for cycling and walking for log-linear DRFs.

*Table S1: RR for cycling and walking with and without adjustment for background air pollution concentrations, based on reanalysis of Kelly et al. (2014) (95% confidence intervals in parenthesis). RR are per 11.25 METh/week change in cycling and walking. Log-linear DRF was assumed in these calculations.*

| RR | Cycling | Walking |  |
| --- | --- | --- | --- |
| RR | 0.903 (0.866-0.943) | 0.886 (0.806-0.973) | |
| RR (adjusted) | 0.901 (0.863-0.940) | 0.884 (0.804-0.971) | |

Counterfactual scenario from car transport

In the main analyses we assumed that counterfactual scenario for cycling is to stay at home. As a sensitivity analysis we also repeated the calculation assuming that increasing cycling would occur by changing the mode of travel from car to bike. In such scenario we assumed that the exposure concentration would decrease 20% (based on updated review of exposure studies comparing exposure concentration in bicycle and car (Kahlmeier et al. 2014)). In this scenario the exposure to PM2.5 was still assumed to increase because of the ventilation rate differences between car (rest ventilation rate was assumed) and bike. We also assumed that time spent driving and cycling would be same.

Shape of the DRF for PM2.5

In the main analyses the DRF for PM2.5 was assumed to be linear. As a sensitivity analyses we calculated the results by using the DRFs from (Burnett et al. 2014). Burnett et al. predicted non-linear DRF for PM2.5 air pollution for different diseases. The DRF for stroke was the most non-linear with maximum harm reached around 300 µg/m3 concentrations. We used Burnett et al.’s DRF for stroke as a hypothetical non-linear DRF for all-cause mortality to predict how non-linear PM2.5 DRF would change the results. See Figure S2 below for illustration of both DRFs for PM2.5.

*Figure S2: Comparison of linear and nonlinear dose-response function (DRF) for PM2.5 air pollution. Non-linear DRF (Stroke) was obtained from* (Burnett et al. 2014)*.*

**Results – additional figures and tables**


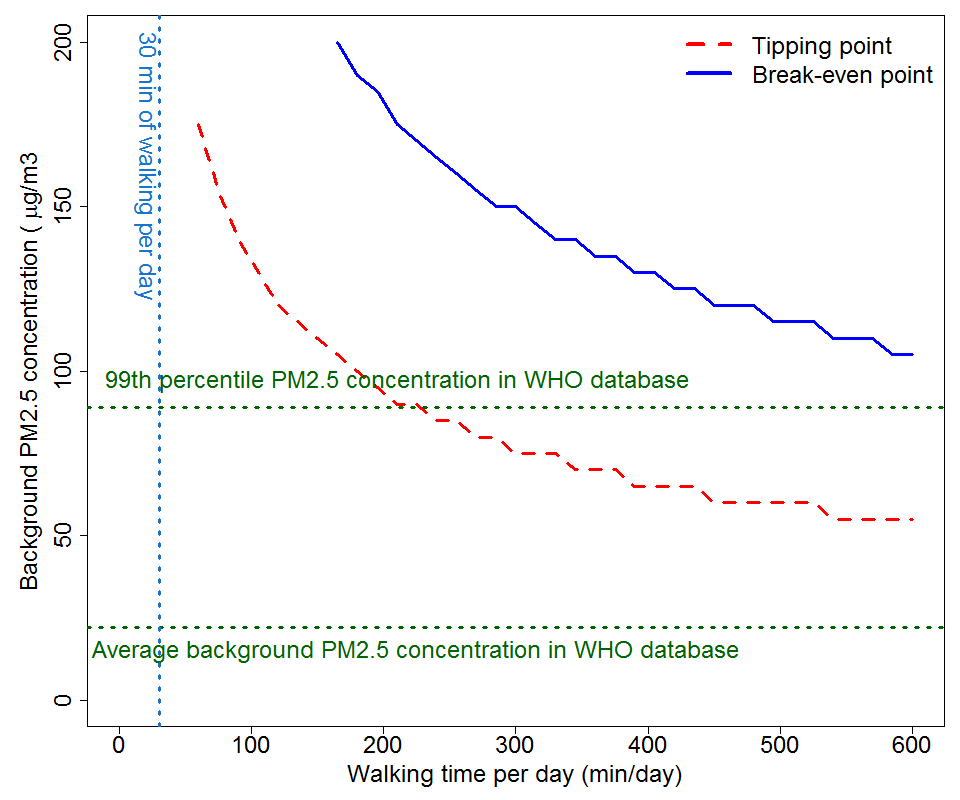


*Figure S3:* *Tipping and break-even points for different levels of walking (red dashed line and blue solid line, respectively) (minutes per day, x-axis) and for different background PM2.5 concentrations (y-axis). Green lines represent the average and 99th percentile background PM2.5 concentrations in World Health Organization (WHO) Ambient Air Pollution Database* (World Health Organization (WHO) 2014)*.*

*Table S2: Tipping and break-even points for cycling in different WHO regions* (World Health Organization (WHO) 2014)*. The average represents the average city in the region and max the city with highest background PM2.5 concentration. PM2.5 concentrations are from WHO* (see article for details)*.*

|  | Average city | | | Most polluted city | | |
| --- | --- | --- | --- | --- | --- | --- |
| Region | PM2.5 (µg/m3) | Tipping point (cycling /day) | Break-event point (cycling /day) | PM2.5 (µg/m3) | Tipping point (cycling /day) | Break-event point (cycling /day) |
| Africa | 26 | 5h | - | 66 | 1h | 3h |
| Americas | 21 | 7h45min | - | 44 | 2h | 6h45min |
| Eastern Mediterranean | 72 | 45min | 2h30min | 117 | 30min | 1h |
| Europe | 37 | 2h30min | 9h15min | 90 | 45min | 1h45min |
| South-East Asia | 43 | 2h | 7h | 153 | 30min | 45min |
| Western Pacific | 39 | 2h15min | 8h30min | 80 | 45min | 2h |

*Table S3: Tipping and break-even points for walking in different WHO regions* (World Health Organization (WHO) 2014)*. The average represents the average city in the region and max the city with highest background PM2.5 concentration. PM2.5 concentrations are from WHO* (see article for details)*.*

|  | Average city | | | Most polluted city | | |
| --- | --- | --- | --- | --- | --- | --- |
| Region | PM2.5 (µg/m3) | Tipping point (walking /day) | Break-event point (walking /day) | PM2.5 (µg/m3) | Tipping point (walking /day) | Break-event point (walking /day) |
| Africa | 26 | - | - | 66 | 6h15min | - |
| Americas | 21 | - | - | 44 | 14h | - |
| Eastern Mediterranean | 72 | 5h30min | - | 117 | 2h15min | 7h45min |
| Europe | 37 | - | - | 90 | 3h30min | 13h15min |
| South-East Asia | 43 | 14h45min | - | 153 | 1h30min | 4h45min |
| Western Pacific | 39 | - | - | 80 | 4h30min | - |

*Figure S4: Break-even point for different DRFs for cycling (see Figure S1). Blue line represent the main analysis, green line break-even point “power 0.25” DRF for cycling and brown line break-even point “power 1.00” DRF for cycling. With the log-linear DRF (power 1.00) the risk of air pollution was always higher than physical activity benefits with the background PM2.5 concentration of 170 µg/m3.*


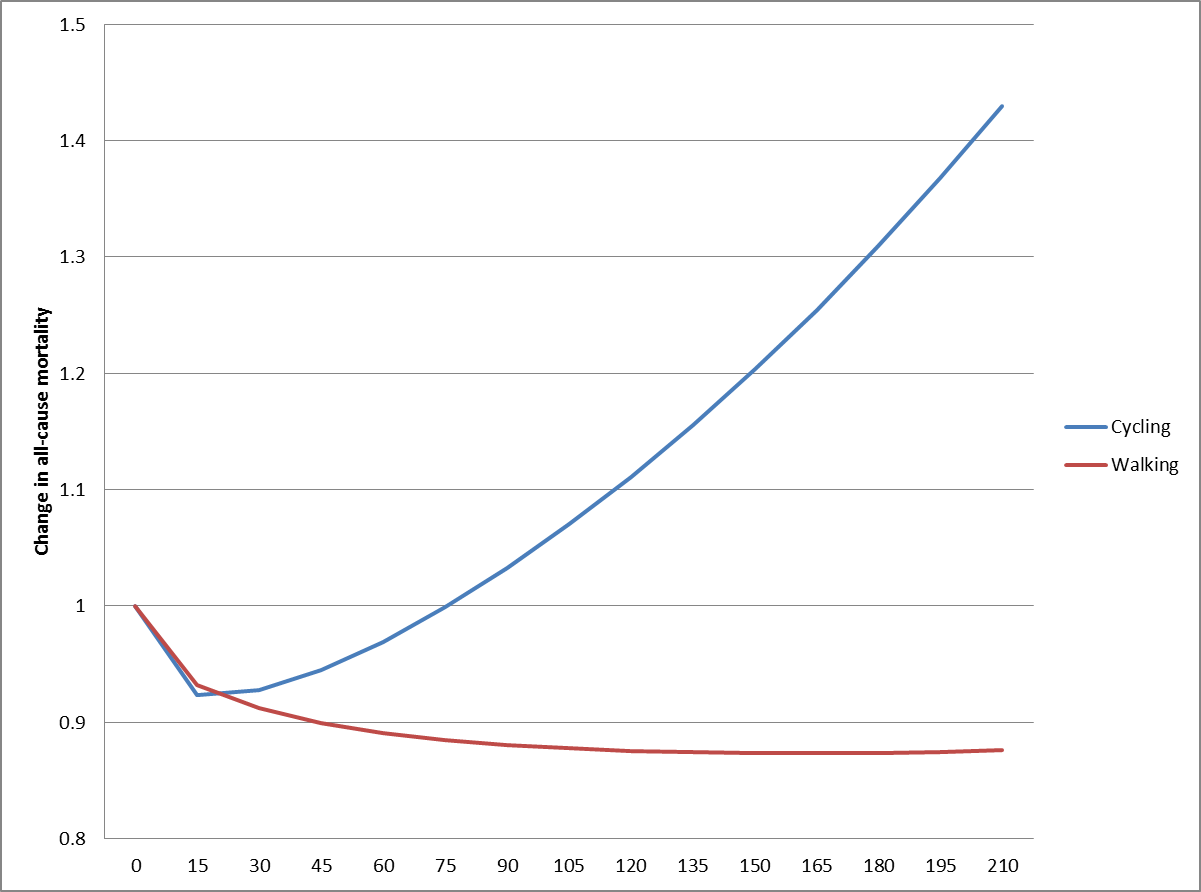


*Figure S5: The change in all-cause mortality for cycling and walking for the background PM2.5 concentration of 100 µg/m3. The x-axis represent cycling and walking time per day (min) and y-axis change in all-cause mortality when both physical activity benefits and air pollution risks were taken into account.*

**References**

Burnett, Richard T, C Arden Pope, Majid Ezzati, Casey Olives, Stephen S Lim, Sumi Mehta, Hwashin H Shin, et al. 2014. “An Integrated Risk Function for Estimating the Global Burden of Disease Attributable to Ambient Fine Particulate Matter Exposure.” *Environmental Health Perspectives* 122 (4): 397–403. doi:10.1289/ehp.1307049.

Kahlmeier, Sonja, Christian Schweizer, David Rojas-Rueda, Mark Nieuwenhuijsen, Audrey de Nazelle, and Olivier Bode. 2014. *Development of the Health Economic Assessment Tools (HEAT) for Walking and Cycling - Meeting Background Document*. Bonn. Germany.

Kelly, Paul, Sonja Kahlmeier, Thomas Götschi, Nicola Orsini, Justin Richards, Nia Roberts, Peter Scarborough, and Charlie Foster. 2014. “Systematic Review and Meta-Analysis of Reduction in All-Cause Mortality from Walking and Cycling and Shape of Dose Response Relationship.” *The International Journal of Behavioral Nutrition and Physical Activity* 11 (1): 132. doi:10.1186/s12966-014-0132-x.

World Health Organization (WHO). 2014. “Ambient Air Pollution Database.” http://apps.who.int/gho/data/view.main.AMBIENTCITY2014?lang=en.
